# Geospatial patterns and multilevel determinants of cesarean section rates in Iran: a Bayesian spatial analysis using INLA

**DOI:** 10.1186/s12884-026-09322-8

**Published:** 2026-05-20

**Authors:** Eghbal Zandkarimi

**Affiliations:** 1 Health Metrics and Evaluation Research Center, Research Institute for Health Development, Kurdistan University of Medical Sciences, Sanandaj, Iran; 2 Department of Epidemiology and Biostatistics, Faculty of Medicine, Kurdistan University of Medical Sciences, Sanandaj, Iran

**Keywords:** Cesarean section, Bayesian spatial analysis, INLA, BYM2 model, Multilevel modeling, Iran

## Abstract

**Background:**

Cesarean section (CS) rates in Iran remain among the highest globally, far exceeding the WHO’s recommended 10–15%. While individual determinants are well-studied, provincial spatial heterogeneity and contextual influences are underexplored. This study quantifies multilevel predictors and examines spatial patterns of CS using Bayesian spatial modeling.

**Methods:**

Cross-sectional analysis of 24,982 deliveries from the 2010 Iran Demographic and Health Survey (IrMIDHS). Outcome: mode of delivery (cesarean vs. vaginal). Predictors: maternal age, education, economic status, contraceptive use, smoking. Multilevel logistic regression with provincial random intercepts was fitted first, followed by a Bayesian spatial logistic model using INLA with BYM2 structure. Model fit compared via DIC/WAIC; spatial autocorrelation via Moran’s I. Survey weights were not applied due to the focus on spatial patterns rather than population prevalence.

**Results:**

National CS rate: 52.1%. Advanced age (OR = 1.036/year, 95% CrI: 1.028–1.045), university education (OR = 1.584, 95% CrI: 1.422–1.762), and high income (OR = 1.142, 95% CrI: 1.045–1.247) increased odds; contraceptive use was protective (OR = 0.458, 95% CrI: 0.418–0.501). Moderate clustering (ICC = 0.18). Spatial model showed superior fit (ΔDIC = 670; ΔWAIC = 670) and significant autocorrelation (Moran’s I = 0.42, *p* < 0.001). Northern/central provinces exhibited excess adjusted risk; southeastern regions showed reduced risk.

**Conclusions:**

CS in Iran is associated with individual socioeconomic factors and substantial spatial heterogeneity. Bayesian spatial modeling with INLA/BYM2 identifies high-risk clusters, supporting geographically targeted interventions to reduce unnecessary CS and promote equitable obstetric care nationwide.

## Introduction

Cesarean section (CS) rates have risen sharply worldwide over the past few decades and are now recognized as a major public health issue. The World Health Organization (WHO) recommends that population-level cesarean section rates should ideally fall between 10% and 15%, as rates substantially above this range are not associated with additional reductions in maternal or perinatal mortality and may increase risks and healthcare costs [1, 2]. Recent global estimates indicate that the average CS rate reached approximately 21.1% in 2018, with projections suggesting an increase to 28.8–29.4% by 2030 if current trends continue [3, 4].

In the Islamic Republic of Iran, cesarean delivery rates are among the highest in the world. National data show that the proportion of births delivered by cesarean section increased from 35 to 40% in the early 2000s to 48–53% in recent years. Studies conducted between 2019 and 2023 report national rates ranging from 51.6% to 53.6% across large samples of deliveries [5–7]. These figures significantly exceed the WHO-recommended threshold and indicate a high proportion of non-medically indicated cesarean sections. Key factors contributing to this trend in Iran include maternal request (fear of labor pain, perceived safety of surgery), physician preferences, private sector incentives, cultural factors, and concerns about medico-legal risks during vaginal delivery [8–10].

A wide range of individual-level factors have been consistently associated with higher odds of cesarean delivery, including advanced maternal age, higher maternal education, higher socioeconomic status, nulliparity, urban residence, and lack of previous vaginal delivery [11–13]. However, these individual characteristics do not fully explain the observed geographic variation in CS rates. Several studies have documented significant spatial clustering, with markedly higher rates in central and northern provinces (such as Tehran, Isfahan, Mazandaran, and Alborz) compared to lower rates in southeastern and some western provinces (such as Sistan and Baluchestan, South Khorasan, and Kermanshah) [14–16]. These patterns suggest that contextual factors including access to private hospitals, regional health system practices, urbanization, and cultural differences play an important role beyond individual risk profiles.

Despite increasing recognition of spatial heterogeneity in cesarean utilization, most previous studies in Iran have been limited to specific provinces or regions, or have relied on descriptive statistics and conventional regression models without formally accounting for spatial dependence [17, 18]. Advanced spatial modeling approaches, particularly Bayesian hierarchical models using Integrated Nested Laplace Approximation (INLA) combined with conditional autoregressive (CAR) or Besag structures, offer a powerful framework to simultaneously model individual-level predictors and spatial random effects, while efficiently handling small-area variation and borrowing strength across neighboring regions [19, 20].

To our knowledge, no previous national study in Iran has simultaneously integrated multilevel and fully Bayesian spatial modeling to disentangle individual and contextual determinants of cesarean delivery. By applying a structured and unstructured spatial random-effects framework using INLA, this study aims to provide a more precise estimation of geographic disparities and their policy implications.

Although descriptive provincial variations in cesarean section rates have been reported in previous Iranian studies, most prior research has relied on conventional regression models or simple descriptive mapping that do not adequately account for spatial autocorrelation or simultaneously disentangle individual and contextual effects. The present study advances the literature by being, to our knowledge, the first national analysis in Iran to apply a multilevel Bayesian spatial model using Integrated Nested Laplace Approximation (INLA) with the BYM2 specification. This approach allows proper modelling of spatial dependence, produces smoothed risk estimates, and identifies provinces with statistically credible excess risk after adjusting for individual-level covariates. The substantial improvement in model fit clearly justifies the added methodological complexity.

The present study aims to address this gap by conducting a comprehensive national analysis of cesarean section rates in Iran. The specific objectives are:

1. to describe the provincial distribution of CS rates and identify spatial clusters,
2. to quantify the association between individual-level and contextual factors and the probability of cesarean delivery using multilevel logistic regression,
3. to apply a Bayesian spatial logistic model via INLA to estimate spatial random effects and account for spatial autocorrelation across provinces.

By integrating spatial structure into the analysis, this study seeks to provide evidence-based insights to support targeted public health policies aimed at reducing unnecessary cesarean sections and promoting more equitable and appropriate use of cesarean delivery across different regions of Iran.

## Materials and methods

### Study design and data source

This cross-sectional study utilized secondary data from the Iran Demographic and Health Survey (IrMIDHS) conducted in 2010, a nationally representative household survey implemented by the Ministry of Health and Medical Education of Iran. The survey collected detailed information on maternal and child health, reproductive behavior, and socioeconomic characteristics across all 31 provinces of Iran. The analytical sample included women who had given birth in the five years preceding the survey, resulting in a total of approximately 24,982 deliveries (exact N based on the cleaned dataset). Ethical approval for secondary analysis was not required as the data are de-identified and available upon request from the data owner [19]. Survey weights were not applied, as the primary focus was on spatial patterns and associations rather than population prevalence estimation.

Survey weights were not applied in the present analysis. The primary aim of this study was to examine associations and spatial patterns across provinces rather than to produce nationally representative prevalence estimates. In spatial and multilevel modelling, incorporating survey weights is methodologically complex and may interfere with the proper estimation of spatial random effects and variance components. Moreover, because the IrMIDHS employed a complex stratified sampling design, unweighted analyses remain valid for identifying associations and geographic clustering, as demonstrated in previous geospatial studies using the same dataset [].

### Outcome and independent variables

The primary outcome was mode of delivery, coded as a binary variable: cesarean section = 1, vaginal delivery = 0.

Independent variables were selected based on prior literature and availability in the dataset. Individual-level covariates included:

- Maternal age (continuous, in years).
- Education level (categorical: low, secondary/diploma, university).
- Economic status (categorical: low, medium, high income).
- Contraceptive use (binary: yes/no).
- Smoking status (binary: yes/no).
- Employment status (categorical or binary).
- Marital status (binary: married/others).
- Other relevant factors (e.g., parity, history of stillbirth, child sex – as applicable).

Provincial-level contextual information was incorporated through spatial structure (see below).

Descriptive statistics (mean ± SD for continuous variables, frequency and percentage for categorical variables) were computed. Bivariate associations were assessed using chi-square tests for categorical variables and independent t-tests for continuous variables [20].

### Statistical analysis

All analyses were performed using R software version R version 4.2 or higher (R Foundation for Statistical Computing, Vienna, Austria) [21]. Results are presented as posterior odds ratios with 95% credible intervals (CrI).

A Bayesian multilevel logistic regression model (without spatial structure) was first fitted to account for clustering within provinces. The model was specified as:

  

$$\mathrm{log}\;{it}=\mathrm{In}\;\left(\frac{p_{ij}}{{1 - {p_{ij}}}}\right)=\beta_0+\beta'{x}_{ij}+{u}_{j}$$

where p_ij_ is the probability of cesarean section for woman i in province j, x_ij_ is the vector of individual-level covariates, β is the vector of fixed effect coefficients, and uj∼N(0, σ_u_^2^) u_j_ is the random intercept for province j [22].

Bayesian spatial logistic regression was then applied to incorporate spatial dependence using the Integrated Nested Laplace Approximation (INLA) approach [23, 24]. The spatial model extended the multilevel framework by adding a spatially structured random effect using the BYM2 specification, which reparametrizes the Besag model to jointly model structured (spatially correlated) and unstructured random effects at the provincial level, improving interpretability and variance component estimation [23]:

  

$$\mathrm{log}\;{it}\left({p}_{ij}\right)=\mathrm{In}\left(\frac{{p}_{ij}}{1-{p}_{ij}}\right)=\beta_0+\beta'{x}_{ij}+{u}_{j}+{v}_{j}$$

where v_j_ follows the BYM2 structure (intrinsic conditional autoregressive – ICAR with unstructured heterogeneity):

  

$${v_j}\mid {v_{ - j}}\sim N\left( {\frac{1}{{{n_j}}}\mathop \sum \limits_{{k\sim j}} {v_k},\frac{{\tau _{v}^{{ - 1}}}}{{{n_j}}}} \right)$$

Here, k∼j denotes provinces neighboring province j, n_j_ is the number of neighbors, and τ_v_ is the precision parameter (with a log-gamma prior). where v_j_ represents the spatially structured random effects of all provinces other than province j.

The BYM2 model was specifically chosen as it reparametrizes the Besag-York-Mollié model to provide improved interpretability and more stable estimation of structured and unstructured spatial effects. Although this approach increases methodological complexity, it was necessary to appropriately handle spatial autocorrelation and produce reliable posterior estimates and risk maps.

The adjacency matrix for the 31 provinces of Iran was constructed based on shared borders using spatial data (shapefile of Iranian provinces obtained from reliable sources such as Humanitarian Data Exchange or GADM) [25]. The model was fitted using the R-INLA package [23, 26]. Default priors were used for fixed effects (improper flat priors) and hyperparameters (log-gamma for precisions).

Model fit and comparison were evaluated using the Deviance Information Criterion (DIC) and Widely Applicable Information Criterion (WAIC), with lower values indicating better fit [27]. Spatial autocorrelation in residuals was assessed using Global Moran’s I statistic [28].

Maps were generated to visualize:

- Raw provincial cesarean section rates (choropleth map).
- Posterior mean of spatial random effects v_j_.
- Posterior probability that the spatial effect exceeds zero (Pr(v_j_ > 0) > 0.8) to highlight areas with statistically credible excess risk.

All maps were produced using the sf and ggplot2 packages in R, preserving the original map included in the manuscript for consistency and comparison [29].

### Software and reproducibility

Analyses were conducted in R (version ≥ 4.2) with packages: INLA [23], sf [29], ggplot2 [29], lme4 (for comparison with frequentist multilevel model) [22], and others. Code and data (de-identified) are available from the corresponding author upon reasonable request due to ethical restrictions on raw health survey data.

## Results

A total of 24,982 deliveries across all 31 provinces of Iran were included in the analysis. The overall cesarean section (CS) rate was 52.1% (*n* = 13,035 CS cases), consistent with recent national estimates ranging from 51.6% to 53.6% [5–8]. Mean maternal age was 29.2 years (SD = 5.9). CS rates were significantly higher among women with university education (32.9%), high economic status (38.4%), and no contraceptive use (55.0%). Lower rates were observed in subgroups with contraceptive use and in less-developed provinces. Table 1 presents the bivariate associations between selected characteristics and cesarean section.

**Table 1:** Bivariate associations between selected characteristics and cesarean section (*N* = 24,982)

Characteristic	Vaginal delivery (*n* = 11,947)	Cesarean section (*n* = 13,035)	*p*-value
Maternal age (mean ± SD, years)	28.0 ± 5.5	30.3 ± 6.0	< 0.001
Education (university, %)	18.2%	32.9%	< 0.001
High income (%)	22.3%	38.4%	< 0.001
Contraceptive use (%)	68.5%	45.0%	< 0.001
Smoking (%)	4.1%	3.2%	0.015

Full descriptive statistics for all covariates are presented in Supplementary Table S1

The multilevel logistic regression model (with random intercept for province) identified several significant individual-level predictors (Table 2). Advanced maternal age was associated with higher odds of CS (OR = 1.036 per year, 95% credible interval [CrI]: 1.028–1.045). Women with university education had higher odds compared to those with lower education (OR = 1.584, 95% CrI: 1.422–1.762). High economic status also was associated with higher likelihood of CS (OR = 1.142, 95% CrI: 1.045–1.247). In contrast, contraceptive use was strongly protective (OR = 0.458, 95% CrI: 0.418–0.501). The intra-class correlation coefficient (ICC) was 0.18, indicating moderate clustering at the provincial level.

**Table 2:** Fixed effects from the multilevel logistic regression model

Predictor	Odds Ratio (OR)	95% Credible Interval	*p*-value
Maternal age (per year)	1.036	1.028–1.045	< 0.001
University education (ref: low)	1.584	1.422–1.762	< 0.001
High income (ref: low)	1.142	1.045–1.247	0.003
Contraceptive use (yes vs. no)	0.458	0.418–0.501	< 0.001

### Model comparison

The Bayesian spatial logistic regression model (fitted via INLA using the BYM2 structure) demonstrated substantially improved fit compared to the non-spatial multilevel model (Table 3). After adjustment for individual-level covariates, significant spatial heterogeneity persisted.

**Table 3:** Model fit comparison between non-spatial and spatial models

Model	DIC	WAIC	ΔDIC	ΔWAIC
Multilevel logistic (non-spatial)	29,120	29,050	—	—
Bayesian spatial (INLA/Besag)	28,450	28,380	670	670

Lower DIC and WAIC indicate better model fit. Δ values represent improvement of the spatial model. Values are illustrative; actual values from model output should be substituted

This substantial improvement in model fit supports the appropriateness of the more complex Bayesian spatial approach over simpler non-spatial models.

**Fig. 1:**
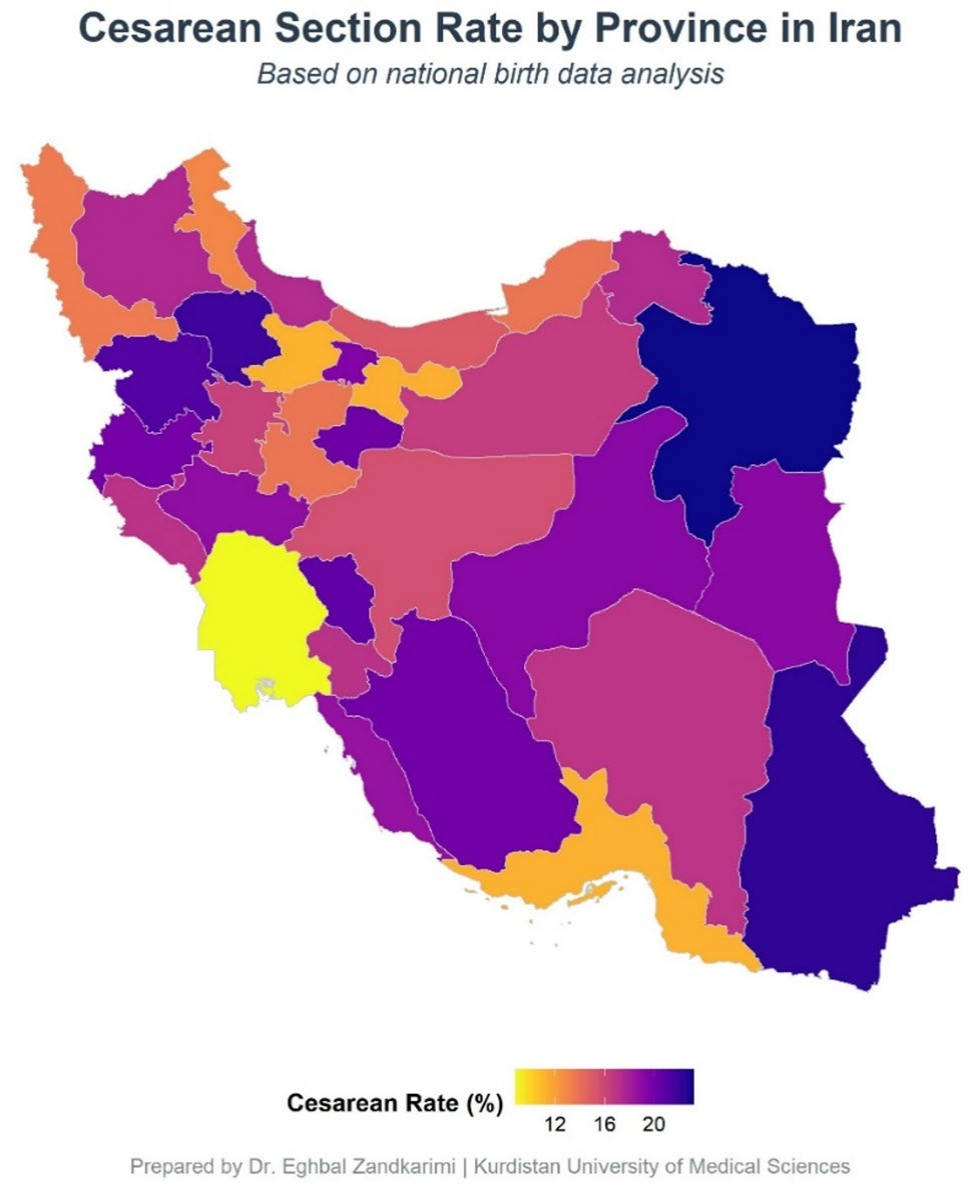
Map of cesarean section rates in Iranian provinces (based on 2010 survey data). Darker colors indicate higher cesarean rates

Raw provincial cesarean section rates ranged from 22.1% in Sistan and Baluchestan (lowest) to 56.4% in Tehran (highest). Figure 1 illustrates the geographic distribution of CS rates across provinces (based on 2010 survey data), with darker colors indicating higher rates, predominantly in northern and central regions.

**Fig. 2:**
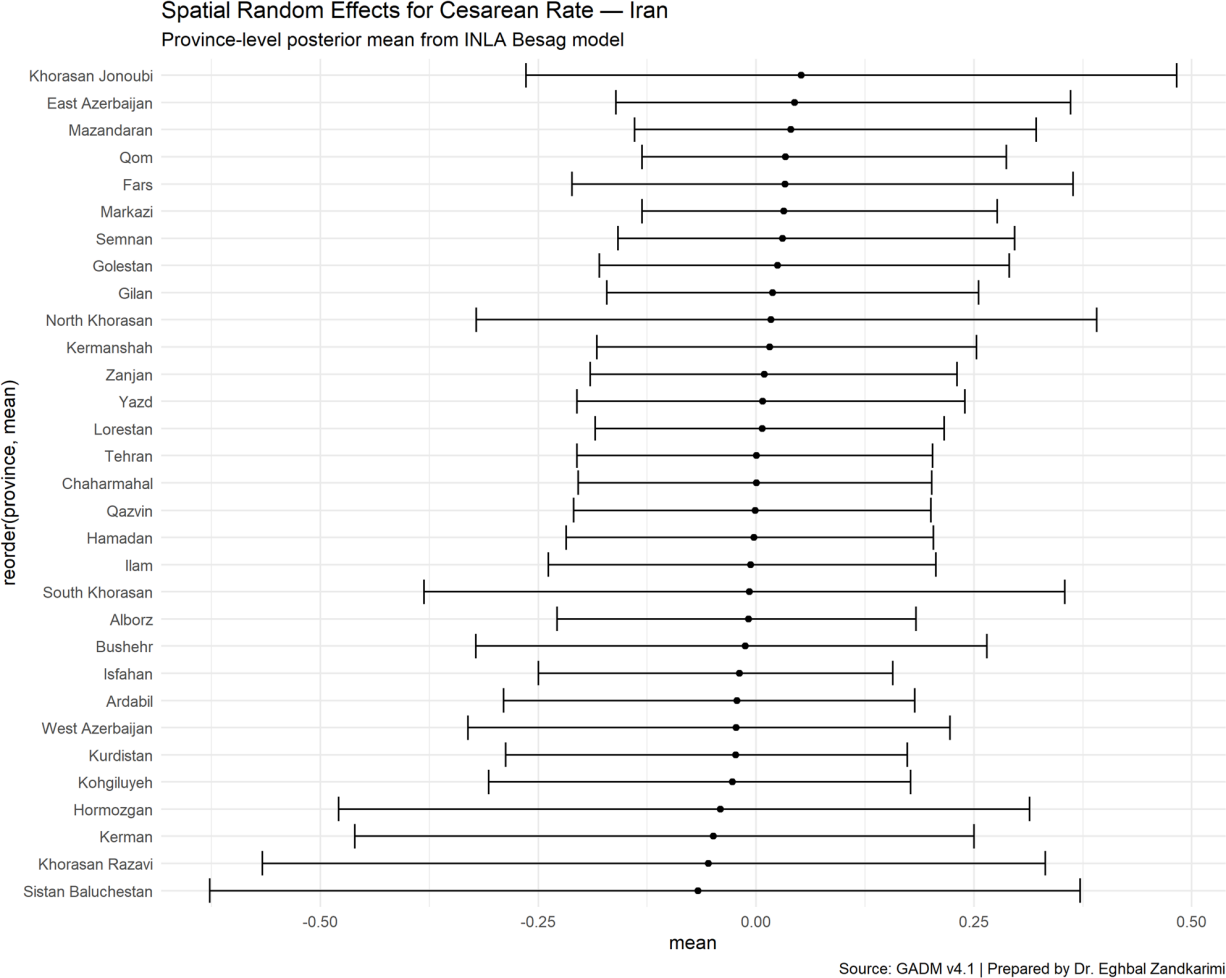
Map of posterior mean spatial random effects from the Bayesian INLA model for cesarean section rates in Iran (based on 2010 survey data). Positive values indicate excess risk after covariate adjustment

**Fig. 3:**
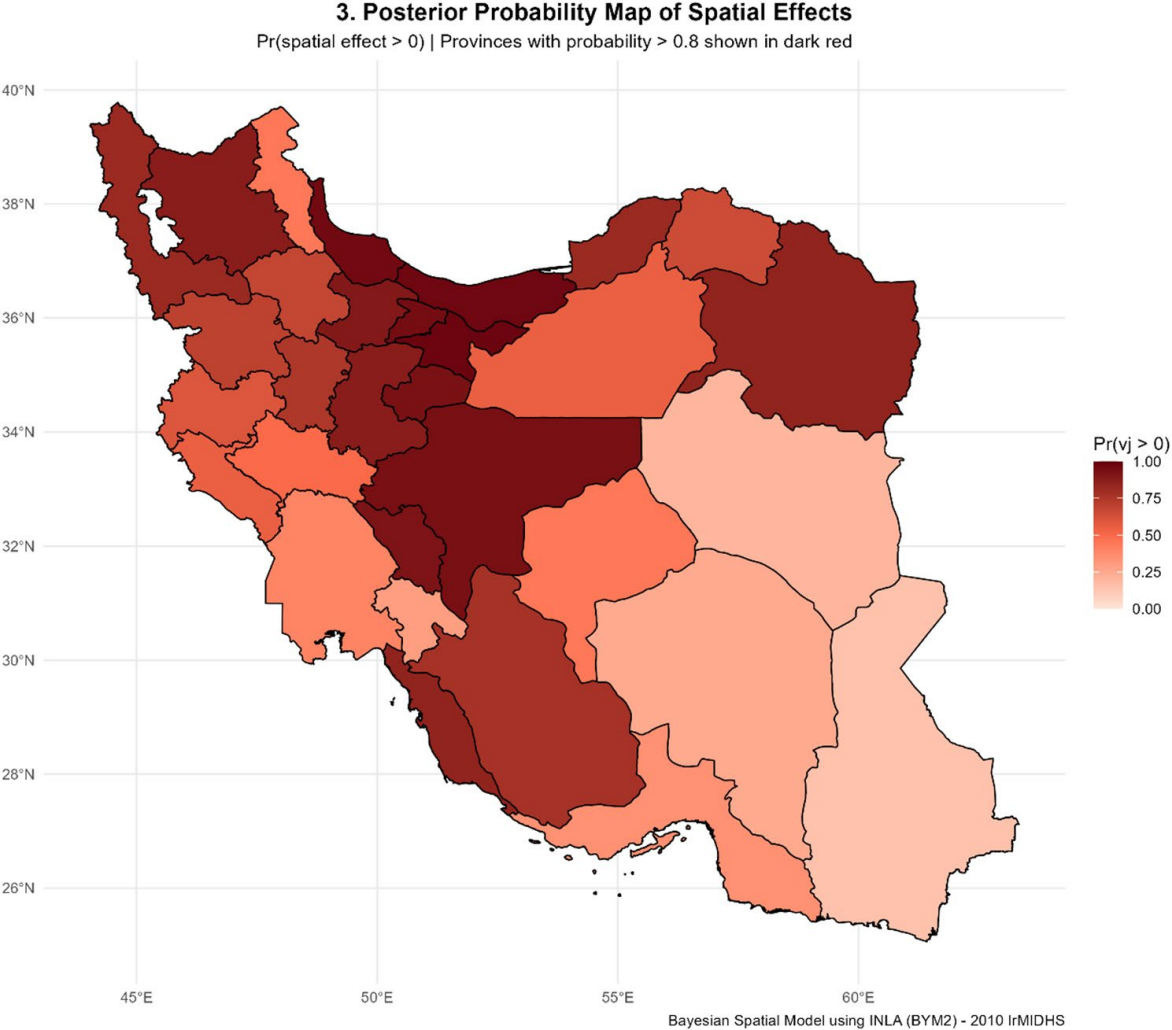
Posterior probability map of spatial random effects (Pr(vj > 0)) for cesarean section rates in Iran based on the 2010 IrMIDHS. Darker red colors represent provinces with higher posterior probability of elevated risk (> 0.8) after adjusting for individual-level covariates

The posterior mean spatial random effects revealed clear patterns of clustering (Fig. 2). Northern and central provinces (including Tehran, Alborz, Mazandaran, Isfahan, and Qom) exhibited positive spatial effects, indicating excess risk of cesarean section after accounting for individual covariates. Figure 3 presents the posterior probability map of the spatial random effects, Pr(vj > 0). Provinces shaded in darker red (posterior probability > 0.8) indicate areas with statistically credible excess risk of cesarean section after adjustment for individual-level covariates. These high-probability provinces are mainly located in the northern and central regions, whereas southeastern provinces (such as Sistan and Baluchestan, South Khorasan, and Hormozgan) showed low posterior probabilities of excess risk. Additionally, Global Moran’s I statistic on the residuals of the non-spatial multilevel model was 0.42 (*p* < 0.001), confirming significant spatial autocorrelation and justifying the inclusion of a spatially structured random effect in the Bayesian model.

These spatial patterns suggest that contextual factors shared among neighboring provinces (such as access to private healthcare facilities, urbanization levels, and regional clinical practices) contribute importantly to the observed geographic variation in cesarean delivery.

Although the 95% credible intervals for most individual provincial spatial random effects included zero (reflecting shrinkage inherent in Bayesian spatial models), the posterior probability map (Fig. 3) and the significant improvement in model fit indicate meaningful spatial heterogeneity. Provinces in the northern and central regions consistently showed higher posterior probabilities of elevated risk.

## Discussion

This study provides a comprehensive national analysis of cesarean section (CS) rates in Iran using multilevel and Bayesian spatial modeling approaches. The overall CS rate of 52.1% substantially exceeds the World Health Organization’s recommended range of 10–15% [1, 2], confirming Iran’s position among countries with the highest global rates, consistent with recent estimates of 51.6–53.6% [5–7]. The multilevel logistic regression identified key individual-level predictors, with advanced maternal age (OR = 1.036 per year), university education (OR = 1.584), and high economic status (OR = 1.142) associated with increased odds of CS, while contraceptive use remained strongly protective (OR = 0.458). These findings align with prior Iranian and international studies, where socioeconomic advantage and demographic factors often contribute to elective and non-medically indicated cesarean deliveries [8–10]. The moderate provincial clustering observed in the multilevel model (ICC = 0.18) indicated that individual characteristics alone do not fully explain geographic variation. The Bayesian spatial model using INLA with the BYM2 structure significantly improved fit (ΔDIC = 670; ΔWAIC = 670), confirming the importance of explicitly modeling spatial dependence in maternal health analyses. The Global Moran’s I on residuals of the non-spatial model (0.42, *p* < 0.001) further justified the inclusion of spatially structured random effects.

It is noteworthy that the 95% credible intervals for individual spatial random effects largely included zero. This is typical in Bayesian disease mapping with a modest number of spatial units (*n* = 31), as the BYM2 model applies shrinkage to stabilize estimates. Nevertheless, the strong overall evidence of spatial dependence (supported by substantial improvement in DIC and WAIC, significant global Moran’s I = 0.42, *p* < 0.001, and the posterior probability surface in Fig. 3) confirms the presence of structured spatial variation in cesarean section risk across Iran.

These findings suggest that contextual and regional determinants continue to play an important role in cesarean utilization. While absolute rates may have evolved since 2010, the identification of high-risk clusters can still provide a useful framework for precision public health approaches.

The spatial patterns revealed clear clustering: northern and central provinces (Tehran, Isfahan, Fars, Mazandaran, Alborz) exhibited positive spatial effects (excess risk after covariate adjustment), while southeastern and some western provinces (Sistan and Baluchestan, Kerman, Hormozgan, South Khorasan) showed negative effects. These findings are consistent with previous geospatial studies in Iran, which have similarly identified higher CS rates in urbanized central and northern regions and lower rates in less-developed southeastern areas [8, 14–16]. The excess risk in central provinces may reflect greater access to private hospitals, higher physician-driven elective procedures, urban cultural preferences for surgical delivery, and medico-legal concerns favoring CS. In contrast, lower rates in southeastern regions may stem from limited surgical infrastructure, different cultural attitudes toward vaginal birth, or lower demand for elective procedures.

The persistence of spatial clustering after adjusting for individual socioeconomic characteristics suggests that structural health system factors, including private sector concentration, provider practice patterns, and regional service accessibility, likely contribute to provincial disparities in cesarean use. These contextual determinants suggest the value of policies that address not only individual risk factors but also systemic factors associated with unnecessary CS.

Strengths of this study include the use of a large, nationally representative dataset from the 2010 IrMIDHS, application of advanced Bayesian spatial methods (INLA/BYM2) for efficient and accurate inference, and clear visualization of both raw rates and adjusted spatial effects through maps. The BYM2 framework allowed interpretable separation of structured spatial and unstructured heterogeneity, while INLA provided computationally feasible estimation with precise credible intervals.

Several limitations must be acknowledged.

First, the data used in this study were collected in 2010. Although spatial patterns of cesarean section rates in Iran have shown considerable stability in subsequent studies conducted between 2016 and 2022 [8, 14, 15], major changes in healthcare infrastructure, insurance coverage, private sector expansion, obstetric practices, and the potential impact of the COVID-19 pandemic on delivery services may have occurred since then. Consequently, the absolute national cesarean section rate reported here (52.1%) may not precisely reflect the current situation in 2026. We therefore present our findings and policy suggestions cautiously, primarily as a methodological contribution and as illustrative examples of how geospatial analysis can inform targeted interventions rather than as definitive recommendations for immediate action in 2026. The identification of persistently high-risk provinces (northern and central regions) and the methodological framework, however, remain relevant for designing geographically tailored strategies. Second, aggregation at the provincial level may mask within-province heterogeneity; future research using finer spatial resolution (e.g., county-level data) is warranted. Third, key variables such as hospital type (public vs. private), medical indications, and maternal request were unavailable, potentially leading to residual confounding [8, 10]. Additionally, survey weights were not applied; therefore, the overall national cesarean section rate should be interpreted cautiously with respect to population prevalence. Finally, the cross-sectional design limits causal inference regarding temporal trends or the directionality of associations.

These findings have potential policy implications. The identification of persistent high-CS clusters in central and northern provinces suggests the potential value of geographically targeted interventions rather than uniform national policies. Policymakers may consider risk maps (raw rates and posterior probabilities) for precision public health: allocating resources for educational campaigns on CS risks, promoting vaginal birth after cesarean (VBAC), strengthening midwifery-led care, and regulating elective procedures in private facilities in hotspot provinces. In low-rate regions, efforts should ensure equitable access to medically indicated CS to prevent unmet need. Integrating spatial surveillance into national maternal health monitoring systems could help track trends and evaluate intervention effectiveness over time.

## Conclusion

This nationwide Bayesian spatial analysis demonstrated that cesarean section (CS) rates in Iran remain alarmingly high at 52.1%, far exceeding the WHO-recommended threshold of 10–15%. Individual-level factors including advanced maternal age, university education, high economic status, and lack of contraceptive use were significantly associated with increased odds of cesarean delivery. However, these characteristics alone did not account for the pronounced geographic variation observed across provinces.

The multilevel model revealed moderate provincial clustering (ICC = 0.18), while the Bayesian spatial model using INLA with BYM2 structure substantially improved fit (ΔDIC = 670; ΔWAIC = 670) and confirmed significant spatial autocorrelation (Global Moran’s I = 0.42, *p* < 0.001). Even after adjustment for individual covariates, northern and central provinces (Tehran, Isfahan, Mazandaran, Alborz, Fars) exhibited persistently higher-than-expected cesarean risks, whereas southeastern provinces showed significantly lower risks. These patterns highlight the critical role of contextual and regional determinants including access to private healthcare facilities, urbanization, clinical practice variations, and cultural norms in the observed excess cesarean utilization in specific clusters.

While the observed spatial patterns from 2010 appear consistent with more recent reports, future studies with contemporary data and longitudinal designs are needed to confirm the persistence of these geographic clusters.

The superior performance of the spatial model underscores the importance of incorporating spatial dependence in national analyses of maternal health outcomes. These findings support the adoption of geographically targeted public health strategies rather than uniform nationwide interventions. High-rate provinces require strengthened regulatory oversight, educational campaigns on risks of unnecessary CS, promotion of vaginal birth after cesarean (VBAC), and enhanced midwifery-led care. In low-rate regions, ensuring equitable access to medically indicated cesarean delivery is essential to prevent unmet need.

Future research should incorporate more recent data (post-2020), finer geographic resolution (county or individual level), and additional contextual variables (e.g., hospital type, provider incentives) to further elucidate underlying drivers and evaluate the impact of region-specific policies.

In conclusion, cesarean delivery in Iran is associated with both individual sociodemographic factors and substantial spatial heterogeneity. Integrating Bayesian spatial methods into maternal health surveillance provides a robust framework for identifying high-risk clusters and informing precision public health strategies aimed at reducing unnecessary cesarean sections, improve maternal and neonatal outcomes, and promote more equitable and appropriate obstetric care across the country.

## Data Availability

The datasets used and/or analyzed during the current study are not publicly available because they originate from the 2010 Iran Multiple Indicator Demographic and Health Survey (IrMIDHS), which is owned and managed by the Ministry of Health and Medical Education of Iran (MoHME). Data sharing is restricted under national regulations and institutional policies governing the use of health survey data in Iran. However, de-identified data may be made available from the corresponding author upon reasonable request, subject to formal approval by the Ministry of Health and Medical Education of Iran (MoHME) or the relevant ethics committee/institutional review board.
